# ENFORCER, internet-based interventions for cardiac arrest survivors: A study protocol for a randomised, parallel-group, multicentre clinical trial

**DOI:** 10.1016/j.resplu.2024.100772

**Published:** 2024-09-18

**Authors:** Lorenzo Gamberini, Paola Rucci, Camilla Dolcini, Martina Masi, Laura Simoncini, Marco Tartaglione, Donatella Del Giudice, Rosa Domina, Andrea Fagiolini, Pamela Salucci, Collaborators, Giovanni Giuliani, Chiara Capozzi, Gianluca Zani, Concetta Lanza, Savino Spadaro, Milo Vason, Maila Mancini, Tommaso Tonetti, Gabriele Melegari, Carlo Pegani, Michele Zuliani, Alice Pravisani, Davide Colombo, Giammaria Cammarota, Rosanna Vaschetto, Giuseppe Ristagno, Nicola Pedroni, Emanuele Rezoagli, Giuseppe Marchese, Fabio Sangalli, Cristina Panzeri, Francesca Verginella, Alberto Cucino, Giulia Roveri, Vittorio Pavoni, Giuliano Michelagnoli, Jacopo Cappellini, Federica Stella, Sonia D’Arrigo, Filippo Sanfilippo, Paolo Murabito, Mariachiara Ippolito, Luca Carenzo, Annalisa Piccolo

**Affiliations:** h Ospedale Infermi – Rimini, Italy; i Ospedale Sant’Orsola-Malpighi - Dipartimento Cardio-Toraco-Vascolare - Bologna, Italy; j Ospedale di Lugo - Lugo, Italy; k Ospedale Morgagni-Pierantoni - Forlì, Italy; l Ospedale Sant’Anna di Ferrara - Rianimazione Universitaria - Ferrara, Italy; m Ospedale Sant’Anna di Ferrara – Rianimazione Ospedaliera - Ferrara, Italy; n Ospedale Santa Maria delle Croci - Ravenna - Anestesia e Rianimazione - Ravenna, Italy; o Ospedale Sant’Orsola-Malpighi - Terapia Intensiva Polivalente - Bologna, Italy; p Ospedale Civile Sant’Agostino – Estense – Baggiovara, Italy; q ASUGI, Azienda Sanitaria Universitaria Giuliano Isontina - 118 Trieste, Italy; r Anestesia e Rianimazione 2 - ASUFC - Ospedale Santissima Maria della Misericordia – Udine, Italy; s UOC anestesia e rianimazione, ASFO, ospedale Santa Maria degli angeli di Pordenone, Italy; t Ospedale Ss. Trinità – Borgomanero, Italy; u Anestesia e Rianimazione Generale, Azienda Ospedaliero Universitaria di Alessandria, Italy; v AOU Maggiore della Carità – Novara, Italy; w Università Statale di Milano – Dipartimento di Fisiopatologia Medico-Chirurgica e dei Trapianti - Milano, Italy; x Anestesia e Rianimazione Neurochirurgica e Generale, ospedale di Circolo – Varese, Italy; y Rianimazione Generale, Ospedale San Gerardo dei Tintori – Monza, Italy; z S.C. Rianimazione e Anestesia Legnano, Ospedale Civile di Legnano – Legnano, Italy; aa Anestesia e Rianimazione, ASST Valtellina e Alto Lario, Sondrio, Italy; ab S.C. Anestesia e Rianimazione, P.O. Manzoni - Lecco, Italy; ac Südtiroler Sanitätsbetrieb - Azienda Sanitaria dell’Alto Adige - Bolzano, Italy; ad Ospedale Santa Chiara - Trento, Italy; ae Anestesia, Rianimazione, Terapia Intensiva e del Dolore – Ospedale di Merano, Italy; af Ospedale Santa Maria Annunziata, USL Toscana Centro – Bagno a Ripoli, Italy; ag Ospedale San Jacopo – Pistoia, Italy; ah Ospedale Santo Stefano – Prato, Italy; ai Centrale Operativa 118 – Venezia, Italy; aj UOC Rianimazione, Terapia Intensiva e Tossicologia Clinica, Policlinico Universitario A. Gemelli-IRCCS, Roma, Italy; ak Azienda Ospedaliero Universitaria Policlinico “G Rodolico - San Marco” - Anestesia e Rianimazione 1 - Catania, Italy; al Azienda Ospedaliero Universitaria Policlinico “G Rodolico - San Marco” - Anestesia e Rianimazione 2 - Catania, Italy; am Anestesia e Rianimazione con Terapia Intensiva Polivalente, Azienda Ospedaliera Universitaria Policlinico Paolo Giaccone, Palermo, Italy; an Dipartimento di Anestesia e Terapia Intensiva, IRCCS Humanitas Research Hospital, Milano, Italy; ao UOC Terapia Intensiva ed Anestesia, Grande Ospedale Metropolitano Bianchi Melacrino Morelli, Reggio Calabria, Italy; a Department of Intensive Care and Prehospital Emergency, Emergency Department, Maggiore Hospital, Bologna, Italy; b Department of Biomedical and Neuromotor Sciences - University of Bologna, Italy; c Neurorehabilitation Unit for Severe Cerebrolesions, Montecatone Rehabilitation Institute, Imola, Italy; d Spinal Unit, Montecatone Rehabilitation Institute, Imola, Italy; e Regional Program Prehospital Emergency 118 - Maggiore Hospital, Bologna, Italy; f Data Protection Office, Bologna Local Health Authority, Bologna, Italy; g Integrated Department of Mental Health and Sensory Organs - University of Siena, Italy

**Keywords:** Out-of-Hospital Cardiac Arrest, Anxiety, Depression, Cognitive dysfunction, Internet-based intervention, Randomized controlled trial, Follow-up studies, Survivors

## Abstract

**Background:**

Out-of-hospital cardiac arrest (OHCA) is a major health concern in Europe, leading to significant morbidity and mortality. Survivors often suffer from cognitive deficits, anxiety, and depression, that affect significantly their quality of life. Current post-discharge care is inconsistent and frequently overlooks subtle but disabling symptoms. The ENFORCER trial aims to significantly enhance the health and quality of life of OHCA survivors by providing a comprehensive, accessible, and user-friendly internet-based lifestyle intervention.

**Methods:**

ENFORCER is a multicentre, parallel group randomized controlled trial involving OHCA survivors aged 18–80 years with cognitive impairment or anxiety/depression measured through validated instruments.

Participants will be randomized 1:1 to the intervention or the control group. The intervention group will receive a one-year program via a secure web application, offering cognitive, emotional, and physical rehabilitation support. The control group will receive standard care.

The primary outcome is the difference in the proportion of patients without cognitive or emotional symptoms between the two groups after one year.

Secondary outcomes include changes in the level of patients’ cognitive and emotional symptoms, quality of life, sleep quality, sexual interest and satisfaction, and caregivers’ burden, quality of life, sleep quality and emotional symptoms in the two groups.

**Discussion:**

The trial addresses the need for consistent post-discharge care, and the timely detection and treatment of cognitive and emotional problems. The internet-based approach allows to potentially reach many patients, ensuring cost-effectiveness and high adherence rates.

The study results could establish a standard for post-OHCA care, improving long-term recovery and quality of life for survivors.

Trial registration.

The trial is registered at clinicaltrials.gov (NCT06395558).

## Introduction

Out-of-hospital Cardiac Arrest (OHCA) significantly contributes to morbidity and mortality in Europe, with rates ranging from 67 to 170 per 100,000 people and an average 8% survival rate after hospital discharge. Approximately half of the survivors experience adverse neurological outcomes.^1^

Recent studies over the past two decades have shown that a significant number of those who survive with favourable neurological statuses, typically classified as Cerebral Performance Category (CPC) 1 or 2, are affected by persistent cognitive deficits, as well as anxiety and depression, which consequently impair their health-related quality of life (HRQoL).^2^, ^3^

Current guidelines from the European Resuscitation Council (ERC) recommend that OHCA survivors be followed up for within three months of discharge to identify and address any psychological or cognitive problems, thereby providing support to both patients and caregivers.^4^

In Italy, the likelihood of surviving OHCA with a positive neurological prognosis is consistent with European data.^5^. However, post-discharge care lacks a consistent approach to assessing cognitive or emotional problems, with follow-up care typically managed by specialists treating the underlying condition responsible for the OHCA (for instance, cardiologists), or in other cases, referring patients back to their primary care physicians. As a result, subtle symptoms may go unrecognized or untreated.

Lifestyle interventions are one possible strategy to support changes in physical and mental health by promoting improvements in cognitive and emotional functioning, physical activity and diet. Such interventions include a variety of programs that include the provision of health education, and cognitive-behavioural therapy (CBT), and may also include practical components such as participation in physical activity or mental exercises.^6^

The implementation of these strategies has been successful in alleviating symptoms in individuals suffering from anxiety and depression,^6^, ^7^, ^8^ and has also been beneficial in improving mild cognitive impairment.^9^ In addition, the use of an internet-based approach to these interventions allows a significant number of patients to be reached while maintaining cost-effectiveness.

In the existing literature, several online projects that have been developed to identify and improve psychological problems through lifestyle changes.^10^, ^11^ Furthermore, these online interventions have shown levels of adherence and effectiveness comparable to traditional treatments that involve a face-to-face approach.^12^, ^13^, ^14^

Nevertheless, there is a lack of specific internet-based lifestyle intervention programs tailored for individuals who have survived OHCA.

ENFORCER (intErnet-based iNterventions FOR Cardiac arrEst suRvivors) is a multicentre randomized controlled interventional trial exploring the effectiveness of a web-based lifestyle intervention on anxiety, depression and cognitive symptoms in cardiac arrest survivors.

## Protocol

The study protocol was drafted according to the SPIRIT (Standard Protocol Items: Recommendations for Interventional Trial) guidelines^15^ and will be reported following the CONSORT (Consolidated Standards of Reporting Trials) guidelines,^16^ together with the CONSORT-EHEALTH extension.^17^

The trial was approved on March 14th, 2024, by the local ethics committee of the sponsor and all participating institutions involved in patient enrolment or treatment (registration number: 74–2024-SPER-AUSLBO). It is registered on clinicaltrials.gov (NCT06395558). The Local Health Authority of Bologna is the sponsor and promoter of the trial, which is funded by a research grant from Fondazione Italian Resuscitation Council ETS (FIRCBRS23-001), a not-for-profit organization dedicated to supporting cardiac arrest survivors and their families (https://www.fondazioneirc.org).

### Aims

The primary aim is to evaluate the effectiveness of internet-based lifestyle interventions on anxiety, depression and cognitive impairment in OHCA survivors with good neurological recovery compared to usual care. The primary outcome is the difference in the proportion of patients without cognitive or emotional symptoms between the two groups one year after enrolment.

The first secondary aim is to evaluate the effectiveness of internet-based lifestyle interventions on HRQoL, behavioural efficiency, and sleep quality in OHCA survivors with good neurological recovery. Outcomes will be assessed at baseline and at 1-, 3-, 6-, and 12-months post-randomization with continuous log-data collection for user engagement.

Other secondary aims include the assessment of caregivers’ HRQoL, anxiety and depression, sleep quality and burden, the validation of the Italian version of the Sexual Interest and Satisfaction Scale (SISS) and the identification of factors associated with anxiety and depression in OHCA survivors with good neurological recovery.

Primary and secondary objectives and outcome measures are listed in Table 1.

**Table 1:** Outcomes and Outcome Measures of the ENFORCER study.

Outcome	Outcome measure
Primary endpoint	
Effectiveness of internet-based lifestyle interventions on anxiety, depression and cognitive impairment	Proportion of patients without cognitive or emotional symptoms (HADS<8 and TICS>31) one year after enrolment
Secondary endpoints	
Effectiveness of internet-based lifestyle interventions on patients’ health-related quality of life, sleep quality, behavioral efficiency, sexual function, and on caregivers’ burden, health-related quality of life, sleep quality and emotional symptoms	Short Form Health Survey 12 (SF-12) Pittsburgh Sleep Quality Index (PSQI) Neurobehavioral rating scale (NRS) Patient Competency Rating Scale (PCRS) Sexual Interest and Satisfaction Scale (SISS) Zarit Burden Inventory (ZBI)
Factors associated with emotional or cognitive impairment	Presence or absence of the cognitive or emotional symptoms at enrollment (t_-1_)
Italian validation of the Sexual Interest and Satisfaction Scale	−

Abbreviations: HADS – hospital anxiety and depression scale; TICS – telephone interview for cognitive status.

### Study design and setting

ENFORCER is an interventional parallel-group randomized controlled trial. Recruited participants will be randomized 1:1 to the intervention or control group using simple computerized randomization obtained at the time of enrolment.

The 38 collaborating institutions will refer potentially eligible patients to the coordinating centre for participation, but also caregivers and self-referral are allowed.

All outpatients who meet the eligibility criteria will be recruited after discharge from hospital, regardless of geographic location.

A trained neuropsychologist, blinded to patient group allocation, will conduct the clinical assessments. All professionals involved in data management and analysis will also be blinded to group allocation. Unblinding will be possible only at the request of the Data Safety and Monitoring Board (DSMB).

### Eligibility criteria

OHCA survivors within 15 days of hospital discharge will be considered eligible for randomization. Inclusion criteria will be age 18–80, no history of severe mental or neurological illness, a good neurological outcome (CPC 1–2) at hospital discharge, Hospital Anxiety and Depression Scale – HADS^18^ score ≥ 8 and/or cognitive impairment (Telephone Interview for Cognitive Status^19^ – TICS score ≤ 31.

Italian language skills and Internet literacy will be required to ensure compliance with treatment and interviews. Prior to enrolment, patients will be asked to give their consent to participate in the study and to have their personal data processed, after an explanation of the study objectives and procedures and an opportunity to ask questions.

Survivors experiencing anxiety, depression, or cognitive impairment will be randomly assigned to the intervention or the control group. For each survivor recruited, a caregiver will also be included in the study, and given access to a parallel account on the web application.

Individuals who do not meet the criteria for anxiety, depression, or cognitive impairment will not be included in the trial and allocated to the treatment groups. However, baseline information will be collected for the secondary analysis to identify factors associated with these conditions after hospital discharge.

### Planned visits

In this study all scheduled visits will be conducted via video web calls to gather data on instruments needing clinician ratings, while self-report questionnaires will be filled out using a web application.

**Fig. 1:**
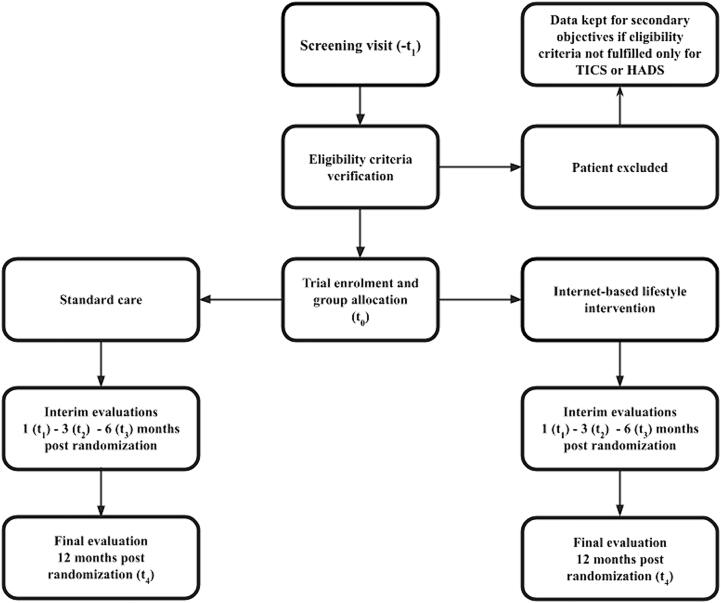
Patients’ flow through the study. Abbreviations: HADS – Hospital Anxiety and Depression Scale, TICS – Telephone Interview for Cognitive Status. Notes: patients not fulfilling the inclusion criteria for HADS or TICS will have their data kept for secondary analyses of the factors associated with anxiety and depression in OHCA survivors with good neurological recovery.

The study procedures are shown in Fig. 1. OHCA survivors who leave the hospital or rehabilitation institute with a good neurological outcome are screened within 15 days of discharge. (visit t-_1_).

Web-based follow-up assessments and self-administered questionnaires are scheduled at 1, 3, 6, and 12 months after randomization. The video web-based face-to-face assessments will focus on those instruments requiring clinical evaluations and monitoring any changes in therapy or hospital admissions. The timeline for participants is shown in Table 2.

**Table 2:** Schedule of enrolment, interventions, and assessments.

Notes: t_-1_ – 0–––14 days from hospital discharge; t_1_ – 1 month post enrolment; t_2_ – 3 months post enrolment; t_3_ – 6 months post enrolment; t_4_ – 12 months post enrolment.

Adherence and intensity of the treatment will be monitored through the number of accesses and the total time spent on the web app. Weekly diaries tracking physical activity (using the Godin Leisure Time Exercise),^20^ social activities, will also be collected. Baseline data, adherence to intervention and safety-related variables are listed in Table 3.

**Table 3:** Data collected and indicators of safety and adherence.

OHCA event and hospital stay-related variables
Age
Sex
Comorbidities
Cause of cardiac arrest
Onset rhythm
Bystander CPR
No flow and low flow time
Presence of STEMI
Targeted Temperature Management
ECMO
Length of ICU stay
Length of hospital stay
Level of education
Employment status before the event
Marital status
Employment status after the event
Weekly diaries
Weekly diaries related to sleep quality, social life, and physical activity
Safety indicators
Number of hospitalizations and their causes during the study period
Death during the study period
Ongoing medications (Sedatives, antidepressants, antipsychotics, opiates, other analgesics)
Ongoing physical therapy
Ongoing psychological therapy
Adherence indicators
Number of accesses to the site
Hours spent on the site
Number of re-readings of information material
Number of exercises completed

Abbreviations: OHCA – out-of-hospital cardiac arrest; CPR – cardiopulmonary resuscitation; STEMI – ST-elevated myocardial infarction; ECMO – Extracorporeal Membrane Oxygenation, ICU – intensive care unit.

The study will utilize several instruments to assess various aspects of patient health and caregiver burden.

These include the HADS and TICS for anxiety, depression, and cognitive impairment;^18^, ^19^ the Short Form 12 (SF-12) for HRQoL;^21^ the Pittsburgh Sleep Quality Index (PSQI) for sleep quality;^22^; the Patient Competency Rating Scale (PCRS) for self-awareness post-brain injury;^23^ and the Neurobehavioral Rating Scale (NRS) for cognitive and noncognitive symptoms.^24^

The SISS,^25^ used for assessing sexual desire and interest post-trauma, will be validated in Italian in this study.

Caregiver HRQoL, anxiety, depression and sleep quality will also be measured using the SF-12, the HADS and the PSQI. Finally, the Zarit Burden Inventory (ZBI)^26^ will be used to assess the impact of the patient’s disability on the caregiver’s daily life.

More details on these instruments are reported in Table 4.

**Table 4:** Outcome measures.

Instrument	Description
Hospital Anxiety and Depression Scale (HADS)	14-item self-report scale used to measure anxiety and depression in patients and caregivers.. A cut-off score < 8 is used to denote the presence of the primary outcome^18^
Telephone Interview for Cognitive Status (TICS)	11-item observer-rated instrument used for cognitive impairment. The cut-off score > 31 is used to denote the presence of the primary outcome^19^
Short-Form 12 (SF-12)	12-item self-report scale used to measure the Health-Related Quality of Life in patients and caregivers. It is an instrument of widespread use in clinical and non-clinical contexts^21^
Pittsburgh Sleep Quality Index (PSQI)	19-item self-report instrument that proved to be a useful, valid and reliable tool for assessing sleep quality.^22^ It is administered to patients and caregivers.
Patient Competency Rating Scale (PCRS)	30-item self-report instrument which asks the subject to rate his or her degree of difficulty in a variety of tasks and functions on a 5-point Likert scale.^23^
Neurobehavioral Rating Scale (NRS)	29-item observer-rated instrument measuring a broad range of cognitive and noncognitive symptoms. It is a structured interview targeting behavioural and cognitive concerns and symptoms, in which the clinician evaluates responses and integrates observational data to characterize the patient’s level of neurobehavioral functioning.^24^
Sexual Interest and Satisfaction Scale (SISS)	6-item self-report scale measuring sexual desire and interest before and after a traumatic event, originally developed for patients affected by spinal cord injury.^25^
Zarit Burden Inventory (ZBI)	A self-report questionnaire of 22-items used to evaluate the burden of care on the caregiver. Its items investigate how a patient’s disability impacts the caregiver’s quality of life, psychological distress, guilt, financial problems, shame, and social and family difficulties.^26^

### Intervention

The program is designed to be self-operating and is delivered through a secure web application created exclusively for this research (https://www.studioenforcer.it/EN).

It is designed to improve cognitive abilities, offer emotional and affective support, and aid in physical rehabilitation.

The one-year program will begin with six months of weekly educational sessions and interactive tasks, followed by six months of occasional reminders to exercise.

To effectively address the most affected areas, patients with cognitive impairment will begin with cognitive enhancement, those with emotional problems will begin with emotional support, and those with both will be randomly assigned to receiving emotional support first, followed by cognitive enhancement or vice versa.

After the initial presentation, all educational and interactive materials will be available for further practice. Physical rehabilitation education will be provided concurrently to ensure a gradual progression towards physical goals.

Caregivers of participants in the study will receive customized education via the web application. Concurrent medical, psychological, or physical therapies are permitted and will be observed at all follow-up stages of the study.

#### Cognitive functions enhancement (3 months)

Cognitive function enhancement educational materials will focus on information about the most common cognitive sequelae after cardiac arrest relative to attention, memory and executive functions, together with suggestions for their management.

Interactive exercises focus on attention (visual item search, go-no go, dual tasks and task switching exercises), memory (learning with immediate and deferred recall of lists of words and texts, immediate learning of visual matrices), executive functions (inhibitory control tasks, logic sequences and problem solving) and working memory (n°-back task, reordering of words lists and mental calculus).

Interactive exercises are graded into three different levels of difficulty, higher levels can be reached only after the correct completion of lower levels, and variability is granted by their continuous modification through artificial intelligence algorithms, to maximize the benefits and possibly increase adherence by avoiding the repetition of the same exercises.

#### Emotional support (3 months)

Emotional support educational material focuses on information about possible emotional-affective sequelae after cardiac arrest and their potential impact on social and personal life, along with strategies for coping. Suggested exercises focus on relaxation, breathing, mindfulness, sleep hygiene, and socialization.

#### Physical rehabilitation (6 months)

Physical rehabilitation materials address the importance of physical activity and proper nutrition in promoting physical and cognitive-emotional improvement. Practical exercises will be presented through pre-registered videos and vignettes.

Rehabilitation will begin with daily strengthening exercises for trunk and arm control and then will focus on aerobic activity, with the aim of achieving at least 150 min of moderate activity and at least 2 muscle-strengthening activities involving all major muscle groups,^27^ within 6 months of enrolment, and maintained for the last 6 months.

Participants will be advised to stop or reduce the intensity of exercises if they feel tired and, to adhere strictly to any physical activity restrictions required by their underlying condition or prescribed by their physicians.

#### Caregiver information

During the first three months, the caregivers in the intervention dyads will receive information about the affective and cognitive problems their relative is likely to experience after a cardiac arrest and about the strategies available to help them.

### Control group

Patients randomized to the control group will be followed up by their primary care physician (usual care). They will be asked to complete the diaries and all the assessments at the same scheduled follow-up visits and as the intervention group.

Throughout the study, participants will be allowed to receive concurrent pharmacological, psychological, or physical treatments. The concurrent use of medications such as antidepressants, anxiolytics, antipsychotics, opiates, and other analgesics, as well as any form of psychotherapy or physiotherapy, will be carefully recorded at the scheduled visits.

### Sample size

The sample size was defined assuming a 25 % difference in the primary outcome (the proportion of patients free of emotional or cognitive disorders at 12 months after enrolment, i.e. HADS<8 and/or TICS>31), i.e. 55 % in the intervention group vs. 30 % in the control group. A total sample size of 96 patients (48 per group) was estimated to be required to reject the null hypothesis of equal proportions in the two groups with 80 % power, an alpha error of 0.05, and a 1:1 randomization. Considering a dropout rate of 30 %, the final sample size was set at 137.

Based on the historical data of the participating centres, an annual flow of 350 patients is expected. According to literature reports that the prevalence of anxiety and depression symptoms is approximately 30 % of OHCA survivors^28^ and cognitive impairment is up to 50 %,^2^ we estimate that about 60–70 % (210–245 patients/year) of patients will be eligible for enrolment, therefore, the full sample size should be reached within 12 months from the beginning of the study. In case of slow recruitment, an extension of the enrolment period up to 24 months will be requested.

### Data collection and statistical analysis plan

Questionnaires and diaries completed by participants will be collected directly via an electronic case report form (eCRF) integrated into a web application. The same application will facilitate data entry for the researcher conducting the observer-rated assessments, using a distinct user interface with unique login credentials. To ensure data quality, the rater will be trained in clinical assessment and completion of the observer-rated questionnaires. Participants will be automatically checked for data completeness, and they will receive regular reminders to ensure timely data entry.

The proportions of patients without cognitive or emotional symptoms 12 months after enrolment will be compared between groups using the z-test. A modified intent-to-treat approach will be used, including in the analysis patients who received at least one the web-based treatment session or standard care. To manage missing/incomplete data and to address a possible selection bias related to the modified intent-to-treat approach we will focus on the possible distribution of the missing data given the observed data. In order to do so, we will determine whether the distribution of patients’ unseen observations at the final visit, given their observations at previous visits and baseline, is different from that seen among the patients who have no missing data. To estimate the intervention effect at the end of the trial, we will make assumptions about how the patterns differ in the two groups of patients. We will then estimate the intervention effect amongst those who do, and do not have, missing data, which has to be averaged, to arrive at the overall estimate of the effect of intervention.^29^

Secondary outcome measures will be compared between the two groups using the *t*-test or Mann-Whitney *U* test according to their frequency distribution.

Factors associated with emotional and/or cognitive dysfunction will be identified using logistic regression models. The dependent variable will be the presence or absence of clinically relevant depression/anxiety (HADS≥8) and/or cognitive impairment (TICS≤31) and independent variables will include patient-related and hospital-related variables. All the patients screened for potential participation in the ENFORCER trial will be included in this analysis. Due to the elevated number of candidate variables to test, the model will be trained in 70 % of the sample using a LASSO (least absolute shrinkage and selection operator) regularization technique to prevent overfitting^30^ and tested in the remaining 30 % of the sample.

Finally, the validation of the Italian version of the SISS will be performed through translation and back-translation of the original version to ensure the semantic equivalence between the two versions. The internal validity of the scale will be assessed by examining the structure and internal consistency of the scale using exploratory factor analysis and Cronbach’s alpha coefficient. The concurrent validity of the SISS with SF-12 and HADS will be investigated using Spearman’s correlation coefficient.

### Harms

Adverse events that may be related to the interventions include harms related to physical activity-related harms and neuropsychiatric deterioration.

Harms related to physical activity may be due to inappropriate or excessive activity for the limitations imposed by the underlying pathology. This possibility is limited by recommendations that patients to strictly follow the indications for their underlying medical conditions.

In case of severe anxiety or depression (HADS≥15) or severe cognitive impairment (TICS≤27) at any time during the study, patients will be referred to the appropriate specialist for a higher level of care.

### Monitoring

An independent Data and Safety Monitoring Board (DSMB) consisting of a biostatistician, a clinician with experience in bioethics, and a stakeholder representative was established before recruitment began. The DMSB will monitor recruitment, adherence to the web-based treatment and safety and will be notified of any adverse events that may occur during the trial.

The main outcomes to be monitored will be the number of patients experiencing serious adverse events (SAEs) and hospitalizations.

### Confidentiality

Pseudonymised data will be collected on the eCRF, while transcoding registries will be kept on separate servers with password-protected access systems. Statistical analysis will be performed on fully anonymised data. The DMSB and regulatory authorities will have access to the database at all times.

### Post-trial care

At the end of the trial, participants in the control group will be given full access to the resources that were initially reserved for to the intervention group.

### Data availability and authorship

Fully anonymized study data will be available in data repositories.

Authorship will be based on the International Committee of Medical Journal Editors (ICMJE) criteria.

### Trial status

The trial (version 3 of the protocol, made available on June 6th, 2024), began on August 5, 2024. The recruitment phase will last one year, followed by another year of follow-up. The study process is expected to be completed by August 2026.

Any changes to the protocol will require the Ethics Committee’s approval. Once these amendments receive approval, they will be documented on Clinicaltrials.gov.

## Discussion

The ENFORCER trial is designed to improve the health and quality of life for OHCA survivors by providing a thorough, easily accessible, and effective intervention programme. This trial focuses on the critical need for reliable post-discharge care, aiming to promptly identify and address any cognitive and emotional problems early on. By using an internet-based approach, the ENFORCER trial has the potential to reach a wide range of patients, ensuring both cost-effectiveness and high adherence to the programme. The results of this trial could be instrumental in setting new standards for post-OHCA care, ultimately improving the long-term recovery and overall quality of life of these survivors.

### Future perspectives

Researchers interested in replicating this study in their own context can request educational resources and exercises. This will facilitate the exploration of the replicability of the study and lay the groundwork for combining the findings in a *meta*-analysis.

Funding

This study is funded by a research grant from Fondazione Italian Resuscitation Council (https://www.fondazioneirc.org/), a not-for-profit organization involved in the assistance and support for cardiac arrest survivors and their families. Fondazione IRC has had only a financial role and has no control over data, results publication and intellectual property of eventual results.

### CRediT authorship contribution statement

**Lorenzo Gamberini:** Writing – original draft, Project administration, Funding acquisition, Conceptualization. **Paola Rucci:** Writing – original draft, Supervision, Methodology, Formal analysis, Conceptualization. **Camilla Dolcini:** Writing – review & editing, Resources, Conceptualization. **Martina Masi:** Writing – original draft, Resources, Conceptualization. **Laura Simoncini:** Writing – original draft, Resources, Conceptualization. **Marco Tartaglione:** Writing – review & editing, Resources, Conceptualization. **Donatella Del Giudice:** Writing – review & editing, Resources, Conceptualization. **Rosa Domina:** Writing – review & editing, Resources, Conceptualization. **Andrea Fagiolini:** Writing – review & editing, Resources, Methodology, Conceptualization. **Pamela Salucci:** Writing – original draft, Project administration, Funding acquisition, Conceptualization.

## Declaration of competing interest

The authors declare that they have no known competing financial interests or personal relationships that could have appeared to influence the work reported in this paper.
